# Large twisted ovarian fibroma associated with Meigs’ syndrome, abdominal pain and severe anemia treated by laparoscopic surgery

**DOI:** 10.1186/1471-2482-14-38

**Published:** 2014-06-24

**Authors:** Antonio Macciò, Clelia Madeddu, Paraskevas Kotsonis, Michele Pietrangeli, Anna Maria Paoletti

**Affiliations:** 1 Department of Gynecologic Oncology, Businco Hospital, Regional Referral Center for Cancer Disease, via Edward Jenner, Cagliari 09121, Italy; 2 Department of Medical Science “Mario Aresu”, University of Cagliari, Cagliari, Italy; 3 Department of Obstetrics and Gynecology, Sirai Hospital, Carbonia, Italy; 4 Department of Obstetrics and Gynecology, University of Cagliari, Cagliari, Italy

**Keywords:** Meigs’ syndrome, Laparoscopy, Hemolytic anemia, Ovarian fibroma

## Abstract

**Background:**

The Meigs' syndrome is a rare but well-known syndrome defined as the triad of benign solid ovarian tumor, ascites, and pleural effusion. Meigs' syndrome always requires surgical treatment. However, the optimal approach for its management has not been sufficiently investigated.

**Case presentation:**

We report a patient with a large twisted ovarian fibroma associated with Meigs’ syndrome, abdominal pain and severe hemolytic anemia that was treated by laparoscopic surgery. This case highlights the difficulties that may be encountered in the management of patients with Meigs’ syndrome, including potential misdiagnosis of the tumor as a malignant ovarian neoplasm that may influence the medical and surgical approach and the adverse impact that Meigs’ syndrome can have on the patient’s condition, especially if it is associated with acute pain and severe anemia. Considering the patient’s serious clinical condition and assuming that she had Meigs' syndrome with a twisted large ovarian mass and possible hemolytic anemia, we first concentrated on effective medical management of our patient and chose the most appropriate surgical treatment after laparoscopic examination. The main aim of our initial approach was preoperative management of the anemia. Blood transfusions and glucocorticoid therapy resulted in stabilization of the hemoglobin level and normalization of the bilirubin levels, which confirmed the appropriateness of this approach. Laparoscopic surgery 4 days after admission enabled definitive diagnosis of the tumor, confirmed torsion and removed the bulky ovarian fibroma, resulting in timely resolution of symptoms, short hospitalization, relatively low morbidity and a rapid return to her social and professional life.

**Conclusions:**

This case highlights the difficulties that may be encountered in the management of patients with Meigs' syndrome, including potential misdiagnosis of the tumor as a malignant ovarian neoplasm that may influence the medical and surgical approach, and the adverse impact that Meigs' syndrome can have on the patient's condition, especially if it is associated with acute pain and severe anemia. The present case suggests that laparoscopic surgery for potentially large malignant tumors is feasible and safe, but requires an appropriate medical and gynecological oncology expertise.

## Background

Ovarian fibromas belong to the group of sex cord-stromal cell tumors and are the most common benign solid tumors of the ovary, accounting for 1–4% of all benign ovarian tumors [1,2]. The most frequent symptoms are abdominal discomfort and pain, but many patients do not experience any specific symptoms. These solid tumors are often difficult to diagnose based on preoperative ultrasonography findings and are commonly misdiagnosed as uterine myomas. They are also sometimes misdiagnosed as malignant ovarian tumors because of accompanying ascites and an increased serum CA-125 level [3]. Ovarian fibromas account for the majority of benign tumors causing Meigs’ syndrome, which is a rare but well-known syndrome defined as the triad of benign solid ovarian tumor, ascites and pleural effusion [4].

Almost all cases of ovarian fibroma can be cured by surgical excision [3]. However, the optimal approach for the management of ovarian fibromas has not been sufficiently investigated. Surgeons may be reluctant to remove the tumor laparoscopically as it can be difficult to safely remove the excised tumor from the abdominal cavity. However, recent advancements in operative instruments and techniques have resulted in laparoscopic surgery becoming increasingly popular among gynecological surgeons.

We report a patient with a large twisted ovarian fibroma associated with Meigs’ syndrome, abdominal pain and severe anemia that was treated by laparoscopic surgery. This case highlights the difficulties that may be encountered in the management of patients with Meigs’ syndrome, including potential misdiagnosis of the tumor as a malignant ovarian neoplasm that may influence the medical and surgical approach and the adverse impact that Meigs’ syndrome can have on the patient’s condition, especially if it is associated with acute pain and severe anemia.

## Case presentation

A 52-year old woman was referred to the Department of Gynecology at Sirai Hospital, Carbonia, Italy with a 4-hour history of abdominal pain that started in the left lower quadrant and subsequently spread to the whole abdomen. She was pale and in obvious discomfort. Her temperature was 39°C, blood pressure was 100/60 mmHg and heart rate was 120 beats/min. Her past medical history was unremarkable except for intermittent episodes of abdominal discomfort and a sensation of abdominal heaviness during the preceding months.

Physical examination revealed a mass that occupied almost the entire abdomen, extending from the lower abdomen to above the umbilicus and restricting mobility. The uterus and adnexae could not be assessed on bimanual pelvic examination.

Pelvic ultrasonography showed ascites throughout the abdomen and an anteverted, enlarged uterus (67 × 54 × 64 mm) with a slightly non-homogeneous myometrial echostructure. The maximum endometrium thickness was 16 mm. The adnexal structures were not recognizable. A non-homogeneous mass (184 × 121 × 184 mm) adjacent to the uterus occupied almost the entire abdomen, extending from the hypogastric region to the epigastric region. No flow was detected in the mass on color Doppler or power Doppler ultrasonography. Contrast-enhanced computed tomography showed a large solid left adnexal mass, ascites and bilateral pleural effusions; a thickened and twisted Fallopian tube with a whirlpool sign was also observed, suggesting adnexal torsion. Contrast-enhanced computed tomography did not show enhancement of the adnexal structures, confirming the hypothesis of torsion and necrosis.

The patient’s hematological parameters at admission are shown in Table 1. She had severe anemia, total bilirubin level of 4.13 mg/dl, indirect bilirubin level of 3.33 mg/dl, elevated CA-125 level and elevated levels of inflammatory markers including the total white blood cell count, percentage of neutrophils, C-reactive protein (CRP) and fibrinogen.

**Table 1:** Patient’s hematological parameters

**Parameters**	**At hospital admittance**	**Before surgery**	**24 hours after surgery**	**48 hours after surgery**	**Hospital discharge**
Leucocytes (10^3^/μl)	19.6	11.0	8.5	7.0	8.6
Neutrophils (%)	88.8	81.9	81.9	79.4	76.5
Lymphocytes (%)	3.8	8.7	11.5	14.3	15.7
Red blood cells (10^6^/μl)	1.99	4.39	4.30	4.08	4.63
Hematocrit (%)	11.3	31.2	29.9	28.5	33.3
Hemoglobin (g/dl)	3.2	10.2	10.3	10.8	10.6
Platelets (10^3^/μl)	177	136	222	156	222
Ca-125 (U/ml)	358	n.v.	n.v.	n.v.	58
C-reactive protein (mg/dl)	20.2	11.3	5.1	2.6	0.4
Fibrinogen (mg/dl)	493	364	330	398	393
D-dimer (ng/ml)	6977	250	177	175	170
Total bilirubin (mg/dl)	4.13	1.31	0.79	1.12	0.93
Direct billirubin	0.77	0.58	0.45	0.50	0.37
Indirect bilirubin	3.33	0.73	0.34	0.62	0.56
Total proteins (g/dl)	4.6	6.4	6.4	6.3	6
Albumin (g/dl)	2.2	3.3	3.2	3.5	3.6

*Abbreviations*: *n.v*. not evaluable.

**Figure 1:**
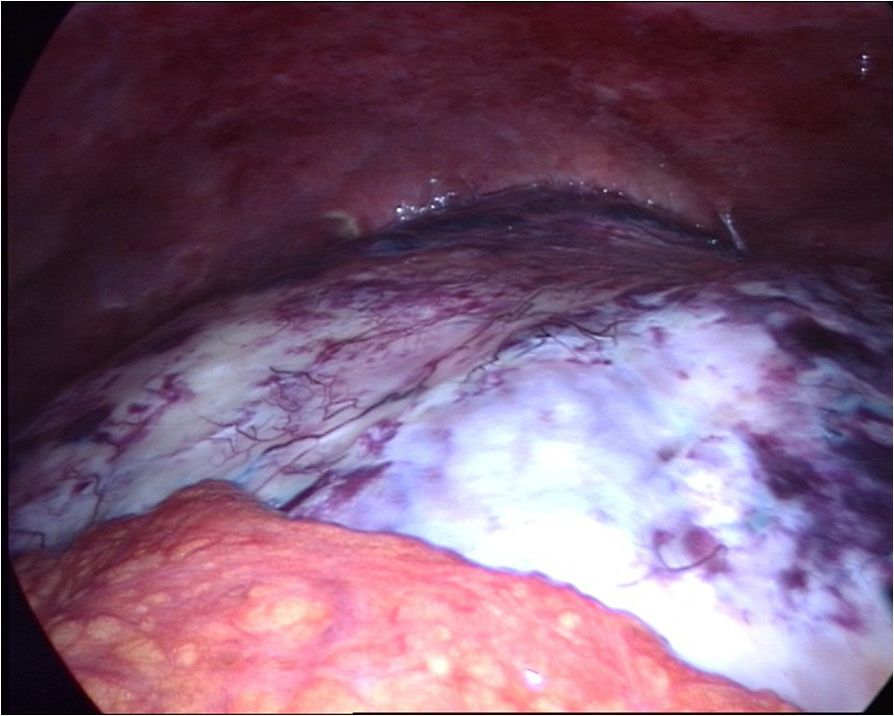
The mass covered by omentum.

**Figure 2:**
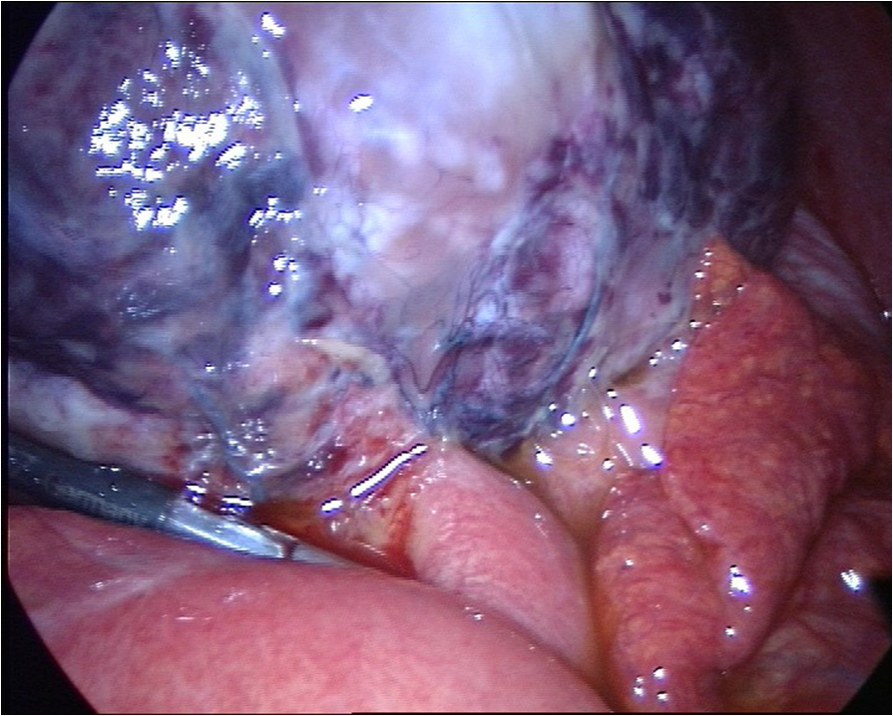
The mass was adherent to parts of the bowel wall.

**Figure 3:**
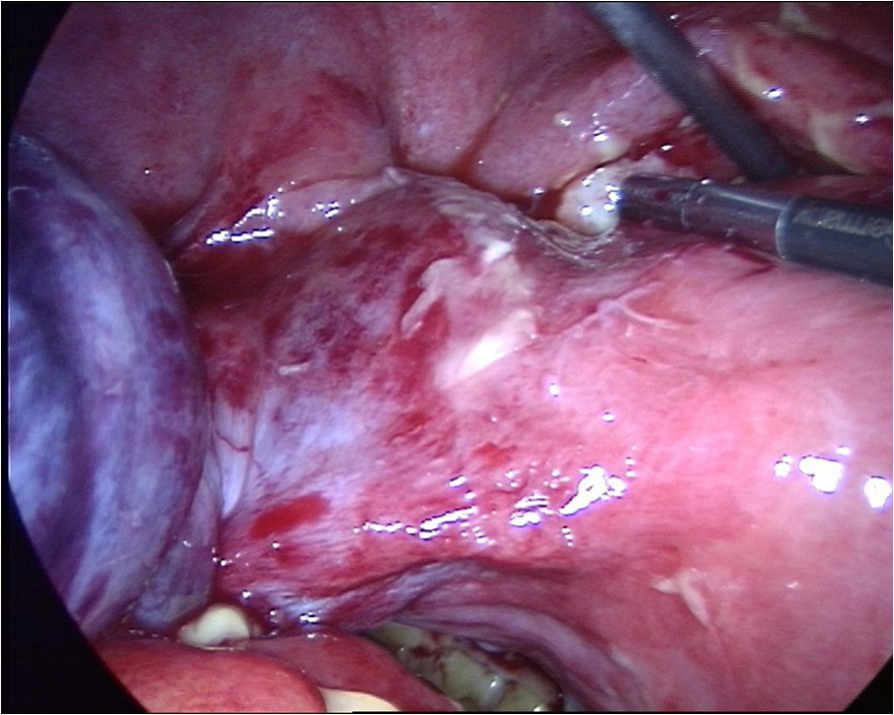
The mass was found to arise from the twisted left adnexa.

**Figure 4:**
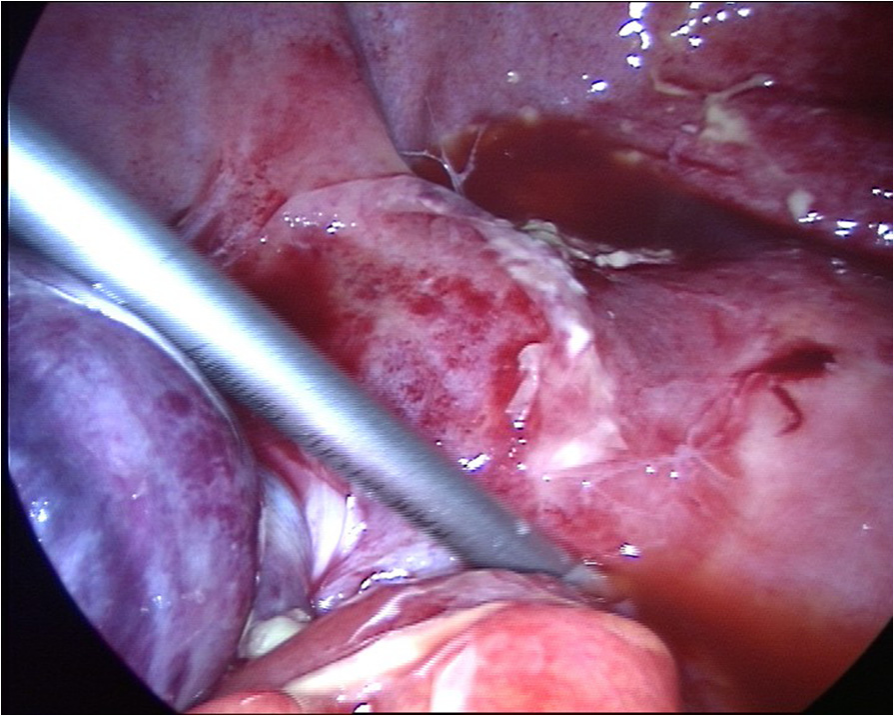
Detail of the twisted adnexa.

Considering the patient’s serious clinical condition and assuming that she had Meigs' syndrome with a twisted large ovarian mass and possible hemolytic anemia, we implemented intensive medical therapy to prepare her for surgery. She received 5 units of packed red blood cells to correct the anemia. In accordance with the accepted international recommendations for the treatment of hemolytic anemia [5,6], she received intravenous methylprednisolone 500 mg/day (Solu-Medrol®; Pfizer, Latina, Italy) for 3 days. She also received subcutaneous low-molecular-weight heparin, antibiotic therapy, albumin infusion and diuretics to avoid progression of her ascites and pleural effusions. Her hematological parameters improved significantly after 3 days, as shown in Table 1. The temporal association between the cessation of hemolysis and glucocorticoid therapy supported our hypothesis of an association between the ovarian tumor and the hemolytic anemia.After improvement of the patient’s condition, we performed laparoscopic surgery on day 4. A port was placed 5 cm above the umbilicus and a pneumoperitoneum of 10–14 mmHg was established and maintained throughout the surgery. Intra-abdominal visualization was achieved using a 10 mm, 0° telescope (Karl Storz, Tuttlingen, Germany) and three 5-mm trocars were introduced under laparoscopic visualization through ports in each lower quadrant and in the suprapubic region. A large amount of ascites was aspirated and a large solid mass was observed. The mass was covered by omentum (Figure 1) and adherent to parts of the bowel wall (Figure 2). The liver, gallbladder, stomach and diaphragm were normal in appearance. The mass was carefully freed from the surrounding adherent structures and was found to arise from the twisted left adnexa (Figures 3 and 4). Malignancy was excluded by intraoperative histological examination without the use laparoscopic power morcellator.

The utero-ovarian ligament, Fallopian tube and infundibulopelvic ligament, which were twisted together, were coagulated using BiClamp LAP forceps (ERBE GmbH, Tubingen, Germany) and the ovarian fibroma was resected using monopolar forceps. In accordance with the recent FDA recommendation regarding the use of internal laparoscopic power morcellation for removal of uterus or uterine fibroids [7], the ovarian fibroma was removed from the abdominal cavity with external morcellation through the supraumbilical port, which was enlarged to approximately 6 cm, with the placement of the “Endopath Dextrus”, to avoid tumor spillage.

The operating time was about 120 min. There was no significant blood loss and no anesthesia-related complications were observed (Table 1). Postoperative pathologic examination of the surgical specimen showed complete hemorrhagic necrosis of an ovarian fibroma with evidence of stromal edema (weight 1,930 g) and a 10-cm long necrotic Fallopian tube. Peritoneal cytology showed inflammation but no malignant cells.

The patient was discharged 5 days after surgery (Table 1) with a small pleural effusion which resolved approximately 2 weeks after discharge. Seven days after discharge she reported a satisfactory return to her normal social and working activities. One month later she had recovered well and was asymptomatic.

## Conclusions

This report presents a rare case of a large twisted ovarian fibroma associated with pain, Meigs’ syndrome and severe hemolytic anemia. This case highlights the complexities associated with the diagnosis and treatment of patients with severe complications of benign ovarian disease. In rare cases, ovarian fibromas are associated with ascites and pleural effusions, which is known as Meigs’ syndrome [4]. This syndrome is usually associated with large fibromas and high CA-125 levels [8,9]. In the present case, making an accurate diagnosis was complicated by a high CA-125 level associated with anemia and high levels of inflammatory markers (CRP and fibrinogen). These associations may indicate advanced ovarian cancer [10] and our patient could potentially have been misdiagnosed with a malignant ovarian tumor. However, a high CA-125 level does not necessarily indicate ovarian cancer [11] and can also be associated with ovarian fibroma and the accompanying ascites [12,13]. In addition, torsion of an ovarian fibroma with subsequent necrosis and inflammation can result in increased serum levels of CA-125 and inflammatory markers [14,15]. The serum CA-125 level does not seem to have high specificity for ovarian malignancy unless it is associated with specific ultrasound findings suggesting malignancy [16]. Our patient did not have ultrasound findings suggesting malignancy and her condition was complicated by severe anemia. Anemia is associated with ovarian malignancy, which is also commonly associated with high levels of fibrinogen and CRP [10]. In our patient, the serum bilirubin levels suggested hemolytic anemia and the hypoechogenic and acellular echographic characteristics of the effusions excluded ongoing hemorrhage. The association between hemolytic anemia and benign ovarian tumors has been recognized for a long time [17] and complete resolution of the hemolysis has been reported after removal of the tumor [18]. Payne et al. [19] reviewed the clinical courses and responses to ovarian cystectomy in 19 patients with hemolytic anemia and benign ovarian tumors reported in the literature up to 1981. Further 11 cases have been reported since then [20-30], bringing the total to 30 cases. The cases reported in the literature reveal the complexity of managing hemolytic anemia associated with ovarian tumors. One of the previously reported patients showed a good response to glucocorticoid therapy and recovered after ovarian cystectomy within 3 weeks of diagnosis [19]. Two patients died prior to surgery, one because of intestinal obstruction and the other because of a transfusion reaction. One patient underwent simultaneous ovarian cystectomy and splenectomy, resulting in complete recovery. Some patients who did not respond well to initial glucocorticoid therapy or splenectomy recovered well after ovarian cystectomy. These different responses to therapy are interesting, but in our opinion it is difficult to understand why the therapeutic options are considered to conflict with one another, especially when the size of the ovarian tumor and the related symptoms indicate that surgical excision is the treatment of choice, as in our patient. Considering the high impact of splenectomy, it may be useful to initially plan glucocorticoid therapy and surgical excision of the ovarian tumor and perform splenectomy only in patients who do not respond to the initial therapy. We therefore first concentrated on effective medical management of our patient and chose the most appropriate surgical treatment after laparoscopic examination. The main aim of our initial approach was preoperative management of the anemia. Blood transfusions and glucocorticoid therapy resulted in stabilization of the hemoglobin level and normalization of the bilirubin levels, which confirmed the appropriateness of this approach. Laparoscopic surgery was performed after stabilization of the anemia.

We consider that a long waiting time before surgery should be avoided, as cure depends on surgical excision of the tumor, and the pathogenic mechanisms that trigger autoimmune responses in patients with benign ovarian tumors are unknown. It seems appropriate to administer medical therapy, including transfusion, to ensure that patients can undergo early surgery. In our patient, this approach was necessary because of the size of the ovarian mass, the ovarian torsion and the resulting severe pain. Furthermore, it is known that large ovarian masses in Meigs' syndrome are often associated with intra-abdominal hypertension up to abdominal compartment syndrome. The chronic development of abdominal hypertension and onset of the abdominal compartment syndrome associated with Meigs' syndrome must be recognized in a timely manner and promptly treated by performing as complete a resection of the pelvic mass as possible [31].

Laparoscopic surgery 4 days after admission enabled definitive diagnosis of the tumor, confirmed torsion and removed the bulky ovarian fibroma, resulting in timely resolution of symptoms, short hospitalization, relatively low morbidity and a rapid return to her social and professional life. Meigs’ syndrome always requires surgical treatment and the laparoscopic approach was successful in this case, with careful handling of the tumor because 1% of well-circumscribed ovarian tumors are malignant [32,33]. In cases of large potentially malignant ovarian masses, laparoscopic surgery may have several potential limitations (tumor rupture, spillage, incomplete resection of lesion and trocar insertion site metastasis). On the other hand, a recent review [34] concluded that there was no good-quality evidence to help quantify the risks and benefits of laparoscopy for the management of early-stage ovarian cancer as routine clinical practice.

In conclusion, laparoscopic surgery for potentially malignant tumors may be feasible and safe [35] but requires an experienced gynecological oncology team and is beyond the expertise of the general surgeon [36]. Then, diagnostic laparoscopy is useful in patients with potentially malignant tumors and laparoscopic tumor resection can be performed also if there are signs of malignancy and removal can be achieved without peritoneal contamination. In the present case, laparoscopic resection was feasible, safe and effective for removal of the large ovarian fibroma.

## Consent

Written informed consent was obtained from the patient for publication of this Case Report and any accompanying images.

## Abbreviations

CRP: C-reactive protein.

## Competing interests

The authors declare that they have no competing interests.

## Authors’ contributions

AM conceived of the study, participated in its design, enrolled the patients and performed the surgical procedures, and drafted the manuscript. CM participated in the study design and coordination and contributed to draft the manuscript. PK participated in the patient assessment, enrolment and surgical treatment. MP participated in the design of the study and coordination. AMP participated in the study design and coordination and helped to draft the manuscript. All authors read and approved the final manuscript.

## Pre-publication history

The pre-publication history for this paper can be accessed here:

http://www.biomedcentral.com/1471-2482/14/38/prepub
